# Operation of the Australian Store.Synchrotron for macromolecular crystallography

**DOI:** 10.1107/S1399004714016174

**Published:** 2014-09-30

**Authors:** Grischa R. Meyer, David Aragão, Nathan J. Mudie, Tom T. Caradoc-Davies, Sheena McGowan, Philip J. Bertling, David Groenewegen, Stevan M. Quenette, Charles S. Bond, Ashley M. Buckle, Steve Androulakis

**Affiliations:** a Monash eResearch Centre, Monash University, Clayton, Victoria 3800, Australia; b Macromolecular Crystallography, Australian Synchrotron, 800 Blackburn Road, Clayton, Victoria 3168, Australia; c Department of Biochemistry and Molecular Biology, Monash University, Clayton, Victoria 3800, Australia; d Library, Monash University, Clayton, Victoria 3800, Australia; e School of Chemistry and Biochemistry, The University of Western Australia, 35 Stirling Highway, Crawley 6009, Western Australia, Australia; f Monash Bioinformatics Platform, Monash University, Clayton, Victoria 3800, Australia

**Keywords:** Australian Store.Synchrotron, data archiving

## Abstract

The Store.Synchrotron service, a fully functional, cloud computing-based solution to raw X-ray data archiving and dissemination at the Australian Synchrotron, is described. The service automatically receives and archives raw diffraction data, related metadata and preliminary results of automated data-processing workflows. Data are able to be shared with collaborators and opened to the public. In the nine months since its deployment in August 2013, the service has handled over 22.4 TB of raw data (∼1.7 million diffraction images). Several real examples from the Australian crystallographic community are described that illustrate the advantages of the approach, which include real-time online data access and fully redundant, secure storage. Discoveries in biological sciences increasingly require multidisciplinary approaches. With this in mind, Store.Synchrotron has been developed as a component within a greater service that can combine data from other instruments at the Australian Synchrotron, as well as instruments at the Australian neutron source ANSTO. It is therefore envisaged that this will serve as a model implementation of raw data archiving and dissemination within the structural biology research community.

## Abstract 2

The Store.Synchrotron service, a fully functional, cloud computing-based solution to raw X-ray data archiving and dissemination at the Australian Synchrotron, is described.

## 1. Introduction   

The deposition and the wide availability of raw diffraction data have far-reaching benefits for the structural biology community (Joosten & Vriend, 2007 ▶; Jones & Kleywegt, 2007 ▶; Androulakis *et al.*, 2008 ▶; Guss & McMahon, 2014 ▶). We recently created TARDIS, a suite of tools for the deposition of X-ray diffraction images in an open-access repository to facilitate their deposition using federated institutional repositories (Androulakis *et al.*, 2008 ▶). Subsequent engagement with the IUCr and closer relationships with the Australian Synchrotron prompted us to develop a new framework for raw X-ray data archiving and dissemination, the Store.Synchrotron service, which is described in this manuscript. This approach aligns well with the policies of both the Australian Synchrotron and the broader Australian research infrastructure, and may provide a model implementation of raw data archiving and dissemination for the global structural biology research community.

This paper goes into detail on several distinct aspects of the Store.Synchrotron service. An outline of each section is provided here to aid in navigation of the paper.

Technical aspects are described in §[Sec sec2]2. In particular, we describe the underlying needs from a technical point of view in §[Sec sec2.1]2.1, the end-to-end user experience in §2.2, details of the design and implementation in §[Sec sec2.3]2.3, a brief summary of usage so far in §[Sec sec2.4]2.4 and practical benefits to the user in §[Sec sec2.5]2.5.

The requirement to open up and publish raw data by the community, publications and funding agencies is discussed in §[Sec sec3]3. The solutions provided by Store.Synchrotron as well as those provided by other similar projects are described. The approach taken by the Australian Synchrotron to encourage and guide users through the process of data publication with rich metadata in concert with publication of their research is given extra emphasis.

The broader context in which Store.Synchrotron operates is examined in §[Sec sec4]4. We provide considerations for other facilities should they want to follow our model in §[Sec sec4.1]4.1. In §[Sec sec4.2]4.2 we examine opportunities for integrating several current and future instrumentation sources into a single service.

## 2. The operation of Store.Synchrotron for the Australian Synchrotron   

The Store.Synchrotron service (https://store.synchrotron.org.au) has been deployed to receive diffraction data from the macromolecular crystallography (MX) beamlines at the Australian Synchrotron (AS) automatically.

When a user starts collecting data at the beamline, the raw diffraction data, images of the crystal in the loop and automated data-processing results are transferred in real time to Store.Synchrotron. This process requires no user intervention and is automatically carried out for all data except commercial proposals.

This system is accessible through any web browser. Data are available immediately at the time of collection to all authorized users. Upon login, users are presented with a listing of data originated by themselves or shared with them by others. Sharing data is possible both with other users of the Australian Synchrotron as well as external parties such as international collaborators. If desired, the data can be opened to the public.

**Figure 1:**
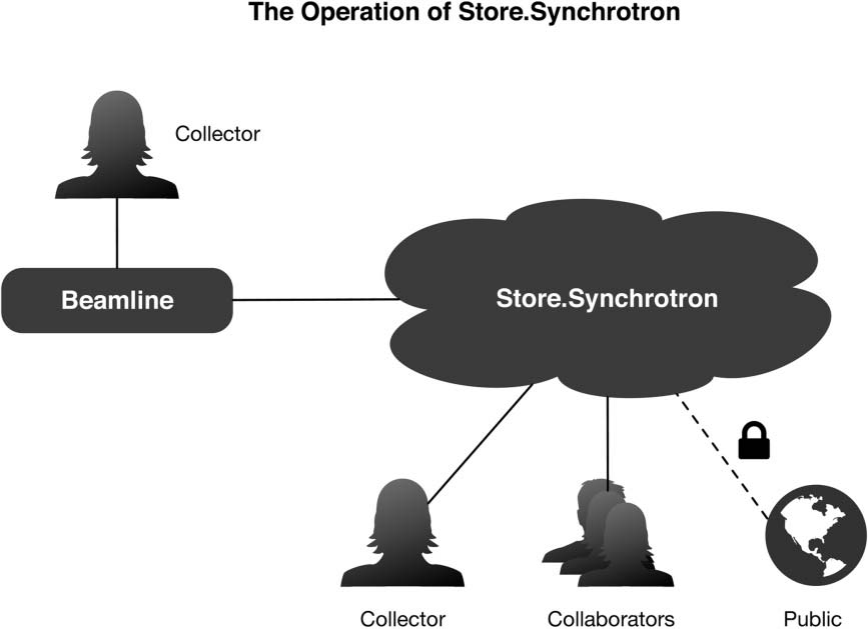
As the user collects data at the beamline, the data are registered with the Store.Synchrotron service. From there, the data are accessible to the collecting user. Data are able to be shared with collaborators and opened to the public.

Fig. 1 ▶ shows a schematic overview of the service and its use and users.

### 2.1. The need for an automated data-management system   

The Australian Synchrotron (AS) has been making a best-faith attempt to store user data, but there had never been a long-term backup of the MX data archive at the AS.

As a result of this limitation and even though other data-access solutions have been available, most users have been taking portable hard drives to the beamlines as this used to be the most practicable solution. Although software is provided at the beamlines to synchronize data to portable hard drives in real time, the use of portable drives creates a series of problems for the user group. Often projects are run by the sole researcher driving the project. There is little standardization in the storage and filing of data. Researchers may choose not to use centralized file servers in their institute for backups and the portable drives may be the laboratory’s sole resource for raw data. After researchers have left it may be difficult to correlate raw frames with data sets and structures. The metadata, which are essential to identify which raw frames match a given structure, may be lost.

In the past there have also been several instances of users contacting the AS for access to raw images relating to data sets that they have lost or when their own media became corrupted. As there was no guaranteed long-term archive, such requests were commonly either cumbersome to fulfil or resulted in disappointment and lost work.

With Store.Synchrotron users now have for the first time a long-term archive of their raw data, including metadata, which can be used to relate raw frames to projects and visits to the Australian Synchrotron.

In addition to the practical need for a robust data archive, new requirements by funding agencies for researchers to have a data-management plan are conveniently solved by a centralized solution that takes care of the data from collection to archiving and/or publishing.

Finally, the availability of affordable service-oriented storage was a key factor that allowed the Store.Synchrotron service to be developed. The data volume for an average experiment is ∼150 GB. With modern storage infrastructure it has become viable to archive all raw data.

### 2.2. The end-to-end user experience   

**Figure 2:**
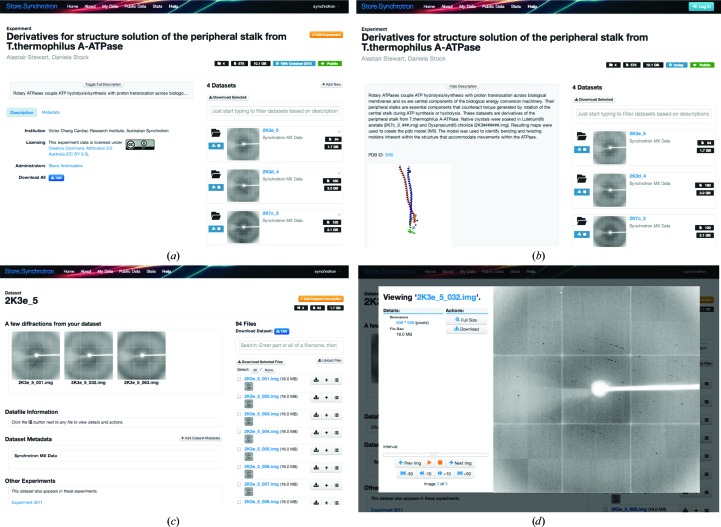
Screenshots of the web interface to the Store.Synchrotron service. (*a*) A public experiment showing a description, license, contact, metrics, download options and a list of the data sets contained within it. (*b*) For public experiments a detailed description with images and links can be shown. (*c*) One data set highlighting a selection of images from the set, optional metadata, metrics, download options and a list of files contained within it. (*d*) Image files can be previewed using the image-viewer overlay.

The default way of accessing data on the Store.Synchrotron service is *via* the web through a web browser. As a user collects a diffraction image it is available on the Store.Synchrotron website within minutes (Fig. 2 ▶).

Data are organized into three tiers, called experiments, data sets and datafiles. An experiment is related to an allocation on the beamline designated by the associated experiment proposal number (EPN; Figs. 2 ▶ *a* and 2 ▶ *b*). The data are then separated into data sets for each full crystal capture (Fig. 2 ▶ *c*). The datafiles, in this case the individual diffraction images, are accessible within the data sets (Fig. 2 ▶ *d*).

The following metadata are automatically provided for each datafile: detector distance, detector signal to noise, direct-beam *x*/*y* positions, exposure time, oscillation range, a preview image and the X-ray wavelength.

For each tier there are download options, such that each experiment, each data set and each datafile can be downloaded as a whole, or a custom selection of data sets or datafiles can be downloaded as a single archive (Fig. 2 ▶).

Some people prefer to access the data *via* other means, most popularly through Rsync/SSH. We developed an SSH/SFTP interface to the service that makes this possible. It is especially useful for retrieving complete and/or large sets of data, as it allows the resumption of interrupted transfers.

While the tiered structure was originally related to the way that the data were stored on disk, these two are completely decoupled in recent versions of the software. Regardless of the underlying structure of data storage, the data are always presented in these familiar tiers.

In addition to diffraction data sets, Store.Synchrotron makes accessible the results of preliminary autoprocessing for each data set. Collection of a single diffraction image triggers automatic indexing by the program *LabelIt* (Sauter *et al.*, 2004 ▶). Collection of a data set triggers automated indexing and full integration and scaling using *XDS* (Kabsch, 2010 ▶) with *XDSme* (https://code.google.com/p/xdsme/) and *AIMLESS* (Evans *et al.*, 2011 ▶). *XDSme* is a Python wrapper (written by Pierre Legrand) that makes use of the programs *XDS* for integration and scaling and *POINTLESS* (Evans, 2006 ▶) to select the space group and unit cell. Statistical descriptors of the data are harvested from the output of the programs and stored in a MongoDB database. All autoprocessing output is able to be monitored in real time and is downloadable, and is often immediately useful in helping live strategic decisions made by a user of the beamline. This is similar to the ISPyB (Delagenière *et al.*, 2011 ▶) system deployed at ESRF and the Diamond Light Source. ISPyB provides real-time monitoring and access to both raw diffraction data and processed results.

Data-collecting users of Store.Synchrotron are able to add other Australian Synchrotron users as collaborators on beamline allocations *via* a separate user-management system. This is used to grant listed collaborators access to an experiment’s data upon login to the service.

While not yet enabled at the Australian Synchrotron, the service supports the creation of a temporary and secret URL that can be shared by users, granting other parties temporary access to the data without the need for an Australian Synchrotron user account. This feature has proved to be especially popular in deployments at other facilities (§[Sec sec4.2]4.2) as a simple and quick access mechanism. This functionality is similar in nature to that found in popular services such as Dropbox.

### 2.3. Design and implementation   

The Store.Synchrotron service is an implementation of MyTardis (http://mytardis.org), an open-source data-management platform. MyTardis began as the TARDIS (Androulakis *et al.*, 2008 ▶; http://tardis.edu.au) diffraction-image repository but has since expanded to suit the storage and organization of a wide range of scientific data. A web portal is provided that allows browsing and access to data and a set of sharing tools to share securely with collaborators as well as release data to the public. The Store.Synchrotron service includes a number of customizations to MyTardis that are also publicly available.

The collection of diffraction data begins at the MX beamlines at the AS. Data are stored in two independent data-storage facilities for redundancy, backup and remote user access. A local four-node EMC Isilon IBM-based network-attached storage (NAS) with a total capacity of 520 TB serves as a temporary holding location for an experiment, while a second copy is transferred off-site to the Store.Synchrotron service. Both raw data and autoprocessing metadata are archived.

The beamline registers its data with Store.Synchrotron using two Python processes on the beamline-control computers. These processes listen to the EPICS control system, triggering actions on the appearance of new data. The first process extracts image-header information, writes out preview images and carries out a SHA-1 checksum for later use in file-integrity verification. Once automatic data processing on a data set is complete, its results are also passed to Store.Synchrotron for display and access. The second process copies the diffraction data to the final networked storage location used for long-term archiving. To ensure that this live process can withstand interruptions in service, communication between the beamline and Store.Synchrotron is mediated using an RQ (http://python-rq.org/) message queue. All communication between the beamline and Store.Synchrotron is encrypted using the HTTPS protocol.

The integrity of the files stored within the Store.Synchrotron service is of the utmost importance. As the data are the results of often nonrepeatable experiments, data are verified *via* checksums at each important step in the data pipeline, starting with data ingestion. The checksum of each file is determined at the time of generation and stored as metadata. Once a file reaches storage, it is verified against the checksum and marked as verified if correct. If a checksum does not match the archived file, it is reported as failed so it can be resent. This process is repeated again when aged data are transferred to tape-backed archival storage. All archived files are re-verified once a month to ensure the ongoing integrity of the service.

Since Store.Synchrotron began operation in August 2013, only eight out of more than 1 700 000 files have been reported as having mismatching checksums. The reason for their failure is unclear, but we speculate that a network interruption during the copy process may have been the cause. This means that the vast majority of data (99.9995%) was transferred successfully and bit-correct in the first transfer attempt. It also demonstrates the value of integrity checking and temporary data-storage processes as these eight files could be re-transferred.

The Store.Synchrotron service receives metadata *via* its RESTful API (https://mytardis.readthedocs.org/en/latest/api.html). The API can be used both for automatic deposition of data and its retrieval. We chose REST because it is a well known and widely used standard for interacting with web applications. By following this standard, we made it trivial for developers to interact with Store.Synchrotron through means other than a web-browser interface. By providing and supporting a standard API, we were able to develop new features and to fix bugs without interrupting the flow of data to and from the service.

Another important technology allowing space- and time-efficient archiving and retrieval of large data sets is SquashFS (http://squashfs.sourceforge.net/). SquashFS is a standard for storing any number of files organized in folders within a single file on disk, similar to a ZIP archive file. It supports compression and random file access as if it were a standard disk file system. Store.Synchrotron transparently combines data sets registered with it into SquashFS files for tape archiving. A single-file retrieval from a tape library is more efficient than retrieving multiple files, and usually the interested party needs access to a number of files from the same data set or a subset of data from one experimental run. Thus, SquashFS makes tape archiving possible in an efficient and less time-consuming manner when compared with archiving the contents of a traditional file system. Furthermore, by retaining a copy of the metadata within each SquashFS file, archives are formed that have potential for use beyond the lifetime of any database or data-access service. It is a genuine long-term storage format.

A copy of recently stored and accessed data, approximately one year’s worth, is stored on fast disk storage co-located with the web server nodes in the cloud for fast on-demand access. Data that are older than a year and have not been recalled manually are safely stored on tape. Thanks to the large capacity and affordability of tape, this data will be available for years or decades to come. While retrieval is not instant, it is just a matter of minutes to recall it back to disk. The user is notified by e-mail and directly within the application once it has been retrieved and can then access it at their convenience.

The variety of storage options available and the storage redundancies are made possible by the complete decoupling of the user-visible file organization and the actual on-disk file organization.

Like a large majority of popular websites, web services and apps, Store.Synchrotron was deployed on a compute cloud. While there are many different cloud offerings by different commercial vendors such as Amazon, Rackspace, Google and Microsoft, it was of particular benefit to us that the Australian Research Cloud Service provided us with our own, publicly funded cloud infrastructure. This infrastructure, based on OpenStack, was created as part of the National eResearch Collaboration Tools and Resources (NeCTAR) Project (http://www.nectar.org.au/research-cloud). The Store.Synchrotron service is currently hosted on a total of 18 cloud servers, 25 TB of cloud-based disk volume storage and 100 TB of tape-backed networked storage operated by Monash University.

Naturally, the question arises why we chose to deploy this service on the cloud rather than on traditional university IT infrastructure. ‘Cloud technology’ is the IT buzzword of the decade, but the benefits behind the hype are real. Fundamentally, the term ‘cloud’ refers to abundant, on-demand compute and storage resources at a data centre operated by a third party. The infrastructure is shared among many parties and efficiencies of scale make available unprecedented elasticity at decreasing cost, on average lower than traditional infrastructure. Cloud technology became popular because it allows anyone to quickly respond to spikes and troughs in demand. To illustrate, initially there are usually a handful of researchers retrieving their data after their visit to the synchrotron. A few days later they might share the data with their collaborators at a different institution. Some researchers will publish their raw data with their publication, and some of those publications will receive a lot of attention. It is easy to imagine a situation where hundreds of crystallographers around the world want to verify and maybe re-analyse the raw data of a particularly controversial or surprising result. Our use of a cloud infrastructure allows us to increase the number of cloud nodes that form the service within minutes whenever we need to respond to spikes in demand. It also allows us to scale the service back again once the excitement about a particular result has faded.

To assist the maintenance and coordination of this service, we have used an orchestration and configuration-management system. For its modernity and ease of use, we decided to use SaltStack (http://www.saltstack.com/). This enables the service to be distributed over a configurable number of cloud nodes, each specializing in a task. For example, different nodes are dedicated to database communications, web requests, load balancing, data processing and integrity checking, and handling incoming data. The number of each type in operation can be adjusted easily to handle higher demand where necessary. Rolling out software updates is a matter of running one SaltStack command on a test set of nodes, and if successful running the same command on the live service itself. The update process takes a few minutes each time and is performed during scheduled downtime of the Australian Synchrotron. The efficient coordination of this service allows frequent updates, including new features and bug fixes, and reduces maintenance times.

There are two other important benefits to running in the cloud, namely more flexibility in choosing software and a more regular, agile schedule for service updates. Store.Synchrotron was developed based on the relatively modern web framework Django, which is popular with startups and other lean enterprises. Such a framework has little tracking in the enterprise IT world and thus also at university IT departments. Using the cloud allowed us to run on a modern OS of our choice (Ubuntu) running a recent version of Python (2.7) and deploy with the help of a modern configuration-management tool (SaltStack). This lean technology infrastructure has allowed agile development of the service and quick reactions to stakeholder input.

Because we are using standards-based tools, our service can be deployed as easily on other compute clouds. The interested reader is invited to run their own service similar to Store.Synchrotron by simply running our three-line deployment script (https://github.com/mytardis/mytardis-salt) on any cloud or virtual machine that they have access to. (Not all of the features mentioned here have been incorporated into the public version at time of writing, but should be available at publication time or soon after.)

### 2.4. Current performance   

At peak beamline use about 20–40 images are ingested per minute, each of 18 MB. Since turning on the service in August 2013 and the first public announcement in November 2013, over 350 experiments were collected. Data pages were viewed over 1600 times and more than 500 downloads were requested.

### 2.5. Benefits from the user perspective   

Protein crystallography has benefited for many years from an open-access community-based approach to the constant development and improvement of data-processing, structure-solution and refinement tools. These improvements are driven by faster data collection owing to advances in synchrotron sources and beamline instrumentation. As these tools evolve, it is often beneficial to revisit projects where the data collected had proven to be intractable at the time. One example of such a project was the X-ray crystal structure of PlyC, a phage lysin with antimicrobial potential. A single data set was collected from a single crystal in late 2005 at APS. Despite numerous attempts, no other crystals were obtained that diffracted to a usable resolution. The single low-resolution data set was indexed and processed and extensive attempts at brute-force and partial molecular replacement (MR) were undertaken. However, without a sufficient molecular-replacement probe a solution was never obtained for the native data set. The scientist who had collected the original data literally shelved the project in a local hard drive in her office. By 2011, she had begun using the refinement program *autoBUSTER* for another project and decided to go back to the original data and see whether the new and improved refinement program could produce better results than were originally obtained for one of the partial MR solutions. The raw data were reprocessed and reassessed with newer data-processing tools, slightly improving the resolution (by the use of CC*; Karplus & Diederichs, 2012 ▶). Refining the partial solution with the re-processed data set in *autoBUSTER* produced electron-density maps that clearly showed unbiased secondary structure. Iterations of manual building and refinement produced the final X-ray crystal structure, which was published in 2012 (McGowan *et al.*, 2012 ▶).

A broadly systematic approach can be taken to processing old data for new results. Data-processing programs are continuously evolving, but usually their increased power is not used against previously collected data. The main reason for this is that raw X-ray diffraction data are in general not available publicly. As the Store.Synchrotron public repository grows, we can envisage a system where as new methods to analyse raw data become available they are used against old data and new merged data sets are produced. This could then be plugged into a system such as *PDB_REDO* (Joosten *et al.*, 2009 ▶; http://www.cmbi.ru.nl/pdb_redo/) to update the structures deposited in the PDB to more modern crystallographic standards.

## 3. Open data for the crystallographic community   

The Australian Synchrotron is the only facility of its type in Australia and is in constant use. As such, the organization has actively encouraged the opening up of the data produced there to encourage efficiencies and reuse. This requires services that enable effective storage, curation and citation.

Provision of services related to making data more easily available are timely, as Australian government research funders have been strengthening their language in this area. The primary funding body in Australia, the Australian Research Council (ARC), recently released their comprehensive *Discovery Projects: Instructions to Applicants for Funding Commencing in 2015*, which expressly addresses the management of data by requiring applicants to

Outline plans for the management of data produced as a result of the proposed research, including but not limited to storage, access and re-use arrangements

(Australian Research Council, 2014 ▶).

Similarly, the other significant funding body in Australia, the National Health and Medical Research Council (NHMRC), developed in partnership with the ARC the *Australian Code for the Responsible Conduct of Research*, which explicitly states that they expect applicants to responsibly manage their data:

Policies are required that address the ownership of research materials and data, their storage, their retention beyond the end of the project, and appropriate access to them by the research community

(National Health and Medical Research Council, 2007 ▶).

These data-management requirements bring Australia more in line with the global research environment and that of international funding agencies such as the US National Science Foundation (NSF) and the UK Medical Research Council (MRC), which both require detailed plans on the storage, archiving, dissemination and enabling of reuse of research data produced as a result of proposed research.

Store.Synchrotron represents the logical extension of a longstanding effort in the macromolecular crystallography community to ensure that satisfactory evidence is provided to support the interpretation of structural experiments. This effort has included unrivalled requirements for validation of data-interpretation processes, including the implementation of cross-validation with *R*_free_ (Brünger, 1992 ▶) and model-building best practice (Kleywegt & Jones, 1997 ▶), and the IUCr’s recommendation of mandatory deposition of published crystal structure coordinates and processed diffraction data (Baker *et al.*, 1996 ▶). An editorial in *Nature* entitled *Research cannot flourish if data are not preserved and made accessible. All concerned must act accordingly* (Editorial, 2009 ▶), describes the challenge to macromolecular crystallography that Store.Synchrotron ensures is met.

Other projects exist to deposit and host openly accessible diffraction data on the web, such as DIMER (https://dimer.uq.edu.au/), the registration-requiring JCSG Repository of Crystallography Datasets (http://www.jcsg.org/) and TARDIS (http://tardis.edu.au/). Store.Synchrotron differs from these services by automatically archiving all diffraction data produced from beamlines at the time of data collection. This act eliminates the need for manual diffraction data deposition by the user as a step towards open crystallographic data.

A European project, PaN-Data (http://pan-data.eu/), has also created infrastructure for the automatic archiving and dissemination of raw diffraction data from facilities such as the Diamond Light Source and the ESRF. This is achieved through a combination of the information-management and real-time data-monitoring system ISPyB (Delagenière *et al.*, 2011 ▶), the metadata store ICAT (http://icatproject.org/) and its data-browsing and access web front-end TopCat (https://code.google.com/p/topcat/). To date, access to data has been private to the users who collect it. Store.Synchrotron extends this functionality by providing both an automatic archival mechanism from the time of data collection and a publishing interface for open access to collected data.

While the benefits of open data are widely recognized and understood within the international crystallographic community – the ability to share, reuse, collaborate on and cite research data – only by removing the obstacles of self-administration and localized handling of data from the responsibility of the researcher themselves can the genuine rewards of open data be realised. Store.Synchrotron simplifies this process by automated capture of the data and related technical metadata and by enabling the publication of the data by single-trigger notification.

The publication of raw research data online can be extreme­ly challenging owing to the inherent financial and technical difficulties in attempting to share large amounts of data over the web. To facilitate this publication process, Store.Synchrotron has developed a publication form to assist researchers in identifying and describing the raw data associated with their experiment and contributing this information towards the creation of a rich descriptive record to enable discovery.

Researchers who have agreed to make their research data publicly available are sent a link to an online submission form which they populate with the details of their experiment including synchrotron proposal number, image prefix details, PDB IDs, title and description of the experiment, a selection of crystallization conditions active in initial crystal production, funding and grant information and a trigger condition to publicly release the data (http://tardis.edu.au/syncpublish). At present, a minimum amount of information is mandatory at the time of publication: a title for the entry, its authors, a short textual description, related publications and associated PDB IDs.

These discipline-descriptive metadata are combined with the technical metadata automatically captured from the synchrotron’s internal databases at the time of data generation. Once the researcher submits the publication form, a librarian at the Monash University Library assesses the record metadata and follows up with the researcher if any additional information or clarification is required. The Australian Synchrotron is also notified so as to collate the data sets for public release. Currently, we mandate that all data are made available under a Creative Commons Attribution 3.0 Australia (CC BY 3.0) license. The assembled data sets (known collectively as an experiment) are sent to the researcher for final approval before release and the experiment link is made publicly available and discoverable *via* the Store.Synchrotron site, with a digital object identifier (DOI) minted for citing the data in research papers.

DOIs exist to give users a persistent identifier accessible by URL and a standard framework for describing them. While a service that hosts data may change or be replaced, the DOI to the data remains the same and is simply updated to resolve to a new URL. Store.Synchrotron handles the minting of DOIs *via* the Australian National Data Service (ANDS). ANDS provide a web service called Cite My Data (http://ands.org.au/services/cite-my-data.html). Once data ready for publication are collated, the service contacts Cite My Data and receives a freshly minted DOI. This information is then passed on to the researcher and can be used for citation in their publications. It is important to note that our service allows the minting of DOIs before the release of data. This is especially useful as researchers will be able to reference the data in their papers before publication.

The principal scientist of the macromolecular beamlines at the Australian Synchrotron contacted recent primary investigators to gauge their interest in sharing their previously collected raw diffraction data. Of the 14 contacted, 11 responded positively offering data for publication, three responded that they were already using TARDIS and two responded unfavourably to the idea. Of those that responded negatively, one was in the process of re-processing old data for publication in a highly competitive field and did not want to share raw data yet and the other did not approve of the project.

**Figure 3:**
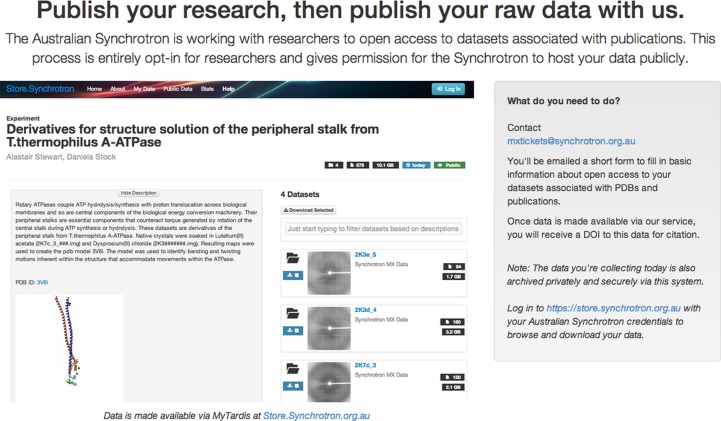
This image shows part of the screensaver on the beamline-control computers.

This initial contact with the Australian crystallographic community has resulted in the publication of 22 raw diffraction data sets from six different research institutions in Australia (https://store.synchrotron.org.au/public_data/). As a result of the largely favourable community reaction, the Australian Synchrotron has amended their user exit interview to include a question asking the user whether they would like to make the data that they have collected during that experiment public in the future and whether they have any data from the past that they would like to release publicly. The Store.Synchrotron service and its public data functionality is advertised on the control computers (Fig. 3 ▶) at both the MX1 and MX2 beamlines.

Store.Synchrotron’s automated capture of metadata and archiving online lowers the traditional barriers to open data for citation in publications. However, current experience has shown room for improved processes in capturing richer metadata about a collection. This is currently being addressed. When releasing data to the public, we see it as crucial to include the richest level of descriptive metadata possible. Given that automated capture of metadata is performed by Store.Synchrotron at the time of diffraction data collection, any step in the scientific method that follows is outside of Store.Synchrotron’s ecosystem and is not automatically included or associated with archived data. Data desired for inclusion when opening raw diffraction data sets are protein structure information, related publication(s) and PubMed IDs.

There are several ways that technology can help to collect a rich level of metadata while minimizing the burden on the researcher. A solution currently under development is a self-serve publish application. Once researchers opt to open data, they will be presented with a web form geared at capturing information from them with minimal effort. This form will have several automated elements embedded such as the entering of a PDB ID or searching for associated PubMed entries, which will in turn trigger the automatic collection and assembly of metadata using the APIs of these external systems. Once the form is submitted, relevant parties are notified (for example, the Australian Synchrotron and the University Library) for final approval. The result of this process is a new, public entry in Store.Synchrotron assembled from this information and a notification to all involved parties.

The metadata associated with each data set at the time of collection will also increase in the near future. As the AS rolls out an electronic experimental authorization (EA) process, users are able to add more information about the proteins they are studying. This can be used in a developmental user GUI which will allow transfer of this metadata to Store.Synchrotron when the data set is collected. The ability to transfer key information such as the name and source of the protein and its sequence information will make the raw data more useful after release. This will also reduce the amount of work required to ‘complete’ data sets for release, as most key information will already be associated with the raw data files. This makes the storage and transfer of metadata transparent to the user and requires minimal effort on the user’s behalf. All data are secure and the release of data on Store.Synchrotron is an opt-in process, so this system does not risk the security or the competitive edge of user groups over international competitors.

## 4. Store.Synchrotron in a broader context   

The data-management infrastructure of the Australian Synchrotron follows a model that is also being applied to a vast array of raw data from other types of scientific instruments in other facilities. There are plans to expand this approach both within the Australian Synchrotron and more broadly. This model is evaluated for use in the global synchrotron community.

### 4.1. Considerations for the use of the Store.Synchrotron model in other facilities   

If this system is to serve as a model for evaluation by other synchrotrons and instrument facilities around the world, it is important to consider potential points of difficulty that may arise with the application of our approach. Certainly, the most difficult infrastructure requirement for Store.Synchrotron is the expense of storage and compute servers.

When operating a data-intensive service, a significant challenge arises: who will pay to keep these data alive? Commodity cloud providers such as Amazon AWS are not yet a cost-effective solution to storing large amounts of frequently accessed data. Thus, research is still reliant on grant or research-institution support to provide the size, resiliency and long-term archiving guarantees expected by researchers, funding bodies and data custodians alike.

To understand how the Australian Synchrotron is to financially support the operation of Store.Synchrotron, it is useful to explain the recent Australian Government research funding and infrastructure environments. The Australian Government has invested heavily in research infrastructure to ease the significant challenge of research data management. The most recent round of funding under the National Collaborative Research Infrastructure Strategy program began in 2009 and has allocated 312 million AUD over five years. Sub-programmes have been established that focus on key areas such as research data management, discovery and reuse.

Monash University is a partner operator of VicNode (http://www.vicnode.org.au), the local node of the Research Data Service Initiative (RDSI; http://www.rdsi.edu.au). VicNode provides a multi-location, tape-archived, large-volume cloud data-storage service that is utilized for all of the data stored at the Store.Synchrotron service. The storage initially provided to Store.Synchrotron by VicNode is over 100 TB in size and is a combination of disk and tape, with an off-site backup. This allocation is expected to grow in the future. Similarly, Store.Synchrotron’s servers have been provided by the NeCTAR compute cloud, of which we have a received merit allocation sufficient to run 64 concurrent servers.

The NeCTAR and RDSI programs waive the capital and operational cost of storage and servers in the near-term. We acknowledge that the funding environment in which our solution operates may not have equivalents elsewhere and so the cost of sophisticated storage infrastructure could remain prohibitively expensive for some. Indeed, the Australian funding environment may also change. To prepare for the long-term operational cost of storage, we also took steps to reduce the amount of data stored *via* the use of data compression and a cheaper, tape-based storage infrastructure for older data (as discussed in §[Sec sec2.3]2.3). The use of a free open-source data-management platform, MyTardis, and its simple deployment on a cloud infrastructure (§[Sec sec2.3]2.3) has done much to reduce the need for software development of data-management infrastructure. MyTardis also provides a level of granularity for captured data about its origin, collector, type and data volume. This in turn can enable decisions such as the the long-term choice to either aggregate expenses at the service level or to recover costs per use, for example as part of research grants.

The minting and ongoing maintenance of digital object identifiers (DOIs) represents an ongoing cost. DOI registration agencies exist worldwide to mint, manage and maintain DOIs, often as a for-profit service. Store.Synchrotron uses the federally run Australian National Data Service (ANDS) as its DOI registration agency. ANDS provide a service that allows the free minting and maintenence of DOIs resolving to Australian research data. Therefore, there is no cost to us to provide DOIs to users.

As discussed in §[Sec sec2.3]2.3, the MyTardis data-management and instrument-integration application is free, open-source and able to be deployed on a compute cloud or web server in an automated fashion. As all instrument facilities have varying data-storage practices, some software-development work almost certainly needs to take place per instrument integration. The integration of both macromolecular beamlines at the Australian Synchrotron with Store.Synchrotron took one software developer approximately four weeks. We envision that our service could offer an attractive model elsewhere for raw data deposition, access, archiving and dissemination.

### 4.2. Integration with current and future instrumentation   

Structural biology increasingly harnesses and integrates multiple approaches to attack challenging problems. In addition to protein crystallography, these include electron microscopy, X-ray scattering and multiscale computational modelling (see, for example, Šali, 1995 ▶). This creates a real need for integrated, end-to-end data-management solutions. In the longer term, the development of new-generation techniques such as XFEL will place significant demands on data management. With this in mind, we have developed Store.Synchrotron as a component within a greater service that combines data from over 25 instruments at other facilities, including the Monash University Micro Imaging platform (electron microscopy) and the Monash University Medical Proteomics Facility (mass spectrometry) as well as instruments at the Australian neutron source ANSTO. This work is built upon the MyTardis data-management platform and thus forms a common interface and data-organization strategy.

Having a single service that integrates and manages a wide array of instrument data can lower barriers to the archiving, organization, curation and sharing of multi-modal data sets. We have already seen local cases where use of the Australian Synchrotron’s diffraction data combined with data from cryo-electron microscopy (EM) have spurred major research discoveries. An example is the discovery that murine perforin forms pores on the cell membrane in the reverse orientation to other cholesterol-dependent cytolysins *via* fitting of the X-ray structure of perforin to the EM map (Law *et al.*, 2010 ▶). This was identified using the EM map and validated using binding experiments to the N- and C-termini, which were proposed to be in the lumen of the pore *via* lectin and antibody binding and the GST-fusion tag, respectively.

These services currently exist in parallel to each other, but work is being performed to combine Store.Synchrotron with the other ‘Store.’ services in the MyTardis federation. This would result in a single URL with a single login to browse and combine research data from several different sources. Once this is achieved, researchers will be able to create entries that contain diffraction data sets, microscopy images and other types of data in a combined interface. This would form a public entry that references several different types of data behind a single discovery.

The Australian Synchrotron itself is working towards the integration of other instruments with the Store.Synchrotron service. Currently, only the MX1 and MX2 beamlines are integrated, but work is being performed on integrating IR and SAXS/WAXS, and work on other beamlines is scheduled to follow in 2015.

## 5. Conclusion   

Store.Synchrotron automatically receives and archives raw diffraction data, related metadata and the preliminary results of automated data-processing workflows. This has delivered much-needed functionality for the Australian crystallographic community, such as real-time online data access and sharing and fully redundant secure storage. In addition to handling over 22.4 TB of raw data since its deployment in August 2013, this cloud-hosted, web-accessible service allows rapid, scalable data processing and distribution that would prove to be a lot more resource-intensive using traditional archival solutions. We envisage that this service will serve as a model for operation in the international macromolecular crystallo­graphy and synchrotron science communities.

## References

[bb1] Androulakis, S. *et al.* (2008). *Acta Cryst.* D**64**, 810–814.

[bb2] Australian Research Council (2014). *Discovery Projects: Instructions to Applicants for Funding Commencing in 2015* http://www.arc.gov.au/pdf/DP15/DP15_ITA.pdf.

[bb3] Baker, E. N., Blundell, T. L., Vijayan, M., Dodson, E., Gilliland, G. L. & Sussman, J. L. (1996). *Acta Cryst.* D**52**, 609. 10.1107/S0907444996000388 15299695

[bb4] Brünger, A. T. (1992). *Nature (London)*, **355**, 472–475. 10.1038/355472a0 18481394

[bb5] Delagenière, S. *et al.* (2011). *Bioinformatics*, **27**, 3186–3192. 10.1093/bioinformatics/btr535 21949273

[bb6] Editorial (2009). *Nature (London)*, **461**, 145. 10.1038/461145b 19741658

[bb8] Evans, G., Axford, D., Waterman, D. & Owen, R. L. (2011). *Crystallogr. Rev.* **17**, 105–142.

[bb7] Evans, P. (2006). *Acta Cryst.* D**62**, 72–82. 10.1107/S0907444905036693 16369096

[bb20] Guss, J. M. & McMahon, B. (2014). *Acta Cryst.* D**70**, 2520–2532. 10.1107/S1399004714005185 PMC4188000 25286838

[bb9] Jones, T. A. & Kleywegt, G. J. (2007). *Science*, **317**, 194–195. 10.1126/science.317.5835.194c 17626864

[bb10] Joosten, R. P. *et al.* (2009). *J. Appl. Cryst.* **42**, 376–384. 10.1107/S0021889809008784 PMC3246819 22477769

[bb11] Joosten, R. P. & Vriend, G. (2007). *Science*, **317**, 195. 10.1126/science.317.5835.195 17626865

[bb12] Kabsch, W. (2010). *Acta Cryst.* D**66**, 125–132. 10.1107/S0907444909047337 PMC2815665 20124692

[bb13] Karplus, P. A. & Diederichs, K. (2012). *Science*, **336**, 1030–1033. 10.1126/science.1218231 PMC3457925 22628654

[bb14] Kleywegt, G. J. & Jones, T. A. (1997). *Methods Enzymol.* **277**, 208–230. 10.1016/s0076-6879(97)77013-7 18488311

[bb15] Law, R. H. *et al.* (2010). *Nature (London)*, **468**, 447–451.

[bb16] McGowan, S., Buckle, A. M., Mitchell, M. S., Hoopes, J. T., Gallagher, D. T., Heselpoth, R. D., Shen, Y., Reboul, C. F., Law, R. H., Fischetti, V. A., Whisstock, J. C. & Nelson, D. C. (2012). *Proc. Natl Acad. Sci. USA*, **109**, 12752–12757. 10.1073/pnas.1208424109 PMC3412044 22807482

[bb17] National Health and Medical Research Council (2007). *Australian Code for the Responsible Conduct of Research* https://www.nhmrc.gov.au/guidelines/publications/r39.

[bb18] Šali, A. (1995). *Curr. Opin. Biotechnol.* **6**, 437–451. 10.1016/0958-1669(95)80074-3 7579655

[bb19] Sauter, N. K., Grosse-Kunstleve, R. W. & Adams, P. D. (2004). *J. Appl. Cryst.* **37**, 399–409. 10.1107/S0021889804005874 PMC2808709 20090869

